# NAT10-Mediated N4-Acetylcytidine of RNA Contributes to Post-transcriptional Regulation of Mouse Oocyte Maturation *in vitro*

**DOI:** 10.3389/fcell.2021.704341

**Published:** 2021-07-30

**Authors:** Yuting Xiang, Chuanchuan Zhou, Yanyan Zeng, Qi Guo, Jiana Huang, Taibao Wu, Jiawen Liu, Qiqi Liang, Haitao Zeng, Xiaoyan Liang

**Affiliations:** Reproductive Medicine Center, Sixth Affiliated Hospital of Sun Yat-sen University, Guangzhou, China

**Keywords:** IVM, NAT10, N4-acetylcytidine, post-transcriptional modulation, RNA modification, oocyte

## Abstract

N4-acetylcytidine (ac4C), a newly identified epigenetic modification within mRNA, has been characterized as a crucial regulator of mRNA stability and translation efficiency. However, the role of ac4C during oocyte maturation, the process mainly controlled via post-transcriptional mechanisms, has not been explored. N-acetyltransferase 10 (NAT10) is the only known enzyme responsible for ac4C production in mammals and ac4C-binding proteins have not been reported yet. In this study, we have documented decreasing trends of both ac4C and NAT10 expression from immature to mature mouse oocytes. With NAT10 knockdown mediated by small interfering RNA (siRNA) in germinal vesicle (GV)-stage oocytes, ac4C modification was reduced and meiotic maturation *in vitro* was significantly retarded. Specifically, the rate of first polar body extrusion was significantly decreased with NAT10 knockdown (34.6%) compared to control oocytes without transfection (74.6%) and oocytes transfected with negative control siRNA (72.6%) (*p* < 0.001), while rates of germinal vesicle breakdown (GVBD) were not significantly different (*p* = 0.6531). RNA immunoprecipitation and high-throughput sequencing using HEK293T cells revealed that the modulated genes were enriched in biological processes associated with nucleosome assembly, chromatin silencing, chromatin modification and cytoskeletal anchoring. In addition, we identified TBL3 as a potential ac4C-binding protein by a bioinformatics algorithm and RNA pulldown with HEK293T cells, which may mediate downstream cellular activities. Taken together, our results suggest that NAT10-mediated ac4C modification is an important regulatory factor during oocyte maturation *in vitro* and TBL3 is a potential ac4C-binding protein.

## Introduction

*In vitro* maturation (IVM) of oocytes is an essential technique in clinical practice of assisted reproductive technology and research of reproductive biology (Walls et al., 2015; Saenz-de-Juano et al., 2019). But the quality and developmental competence of oocytes maturated *in vitro* are still suboptimal (Walls et al., 2015; Li et al., 2021). Further investigation on the regulatory mechanisms of oocyte maturation is necessary.

Oocyte maturation is the physiological event that precedes, and is required for, successful fertilization and embryo development. Human and mouse oocytes enter the early stages of meiosis during fetal life and remain arrested at the diplotene stage of prophase I, also called germinal vesicle (GV)-stage, until they are committed to ovulation or atresia. Unlike somatic cells, transcription and translation are uncoupled during different stages of oocyte maturation. The maternal transcriptome is accumulated during the growth phase of oogenesis and transcription ceases in full-grown GV oocytes. When triggered by hormones, GV-stage oocytes complete meiosis I (MI) and advance to metaphase II (MII), accompanied by utilization of the assembled maternal mRNA. Also, oocyte maturation triggers a transition from mRNA stability to instability, leading to actively degradation of approximately 20% of total maternal transcriptome (Su et al., 2007; Ma et al., 2015). Therefore, post-transcriptional regulation underpinning mRNA stability and translation is a key determinant of gene expression during oocyte maturation (Ivanova et al., 2017). However, the detailed mechanism underlying the subtle regulation remains to be unraveled.

Epigenetic modifications of messenger RNA (mRNA) are emerging as an important component of post-transcriptional control over gene expression involving multiple processes including pre-mRNA splicing, mRNA localization, translation initiation, and mRNA degradation (Wang et al., 2014; Edupuganti et al., 2017; Huang et al., 2018). More than 170 types of RNA modifications have been identified and some of them have been reported to participate in various biological processes, including embryo development, tumorigenesis, and the pathogenesis of metabolic syndrome (Ivanova et al., 2017; Stricker et al., 2017; Huang et al., 2018; Tuncel and Kalkan, 2019). It is of great significance to explore the profiles of mRNA modifications during IVM and identify the critical epigenetic event, which can potentially optimize the culture conditions of IVM and achieve better clinical outcomes. Ivanova et al. (2017) found that YTHDF2, a reader of N6-methyladenosine (m6A) modification, is an intrinsic determinant of mammalian oocyte competence and early zygotic development by binding and destabilizing m6A-modified mRNA during oocyte maturation. Another m6A reader YTHDC1 regulates alternative polyadenylation and splicing during mouse oocyte development (Kasowitz et al., 2018). However, limited by inadequate samples and detection methods, the roles of other mRNA modifications in IVM remain unexplored.

Recently, N4-acetylcytidine (ac4C) has been newly identified as an mRNA modification, which is also a key determinant of post-transcriptional regulation (Arango et al., 2018). ac4C was initially detected in the bacterial transfer RNA (tRNA) anticodon (Stern and Schulman, 1978) and subsequently described in eukaryotic serine and leucine tRNAs and 18S ribosomal RNAs (rRNA) (Boccaletto and Baginski, 2021). Arango et al. (2018) utilized transcriptome-wide approaches and found that ac4C is widely distributed within the human transcriptome with many sites in coding sequences. It is also revealed that mRNA acetylation within coding sequences promotes translation and mRNA stability, while ac4C in wobble sites stimulates translation efficiency (Arango et al., 2018). Since modulation of mRNA stability and translation is the critical event during oocyte maturation, whether ac4C modification participates in the regulation of this process remains to be elucidated.

In addition, it has been known so far that ac4C modification within mRNA, tRNA and 18s rRNA of various species are catalyzed by the N-acetyltransferase 10 (NAT10) enzyme or its homologs (Chimnaronk et al., 2009; Ito et al., 2014; Sharma et al., 2015; Jin et al., 2020). In mammals, NAT10 is the only known RNA acetyltransferase (writer) and is responsible for the ac4C production within a broad range of mRNAs (Arango et al., 2018). However, the recognition of ac4C modification and mechanisms mediating downstream processes remain poorly understood. ac4C-binding proteins (readers) which preferentially bind to ac4C and elicit downstream functions have not been reported yet.

In this study, we sought to investigate the role of NAT10-mediated ac4C modification during oocyte maturation *in vitro*. We observed significantly decreasing trends of both ac4C and NAT10 expression from immature to mature oocytes. NAT10 knockdown in GV-stage mouse oocytes resulted in reduced rate of first polar body extrusion. RNA immunoprecipitation (RIP) and High-throughput sequencing using HEK293T cells revealed that the dysregulated genes were enriched in biological processes associated with oocyte maturation, including nucleosome assembly, chromatin silencing, chromatin modification and cytoskeletal anchoring. Our data further indicated that Transducin beta-like protein 3 (TBL3) is a potential ac4C-binding protein, through which ac4C modification modulates downstream cellular activities.

## Materials and Methods

### Animals

All C57BL/6 mice were purchased from GemPharmatech Co., Ltd. (Nanjing, China) and subjected to an adaptation period of one week before the experiments. The mice were housed in the animal facilities with a 12 h:12 h light/dark cycle, controlled temperature (22–24°C) and humidity (50–60%), with free access to water and food. All procedures were approved by the Animal Care and Use Committee of the Sixth Affiliated Hospital, Sun Yat-sen University (Guangzhou, China) (ethical approval number: IACUC-2020120401).

### Oocyte Collection

For the collection of GV-stage oocytes, 4–5 weeks old females C57BL/6 mice were injected intraperitoneally with 10U of pregnant mare serum gonadotropin (PMSG) (Ningbo Second Hormone Factory, Hangzhou, China). Forty six to forty eight hours after PMSG treatment, cumulus-enclosed oocytes were harvested from ovaries by puncturing antral follicles with a sterile needle in M2 medium (Sigma-Aldrich, MO, United States; Cat# M7167). Denuded GV oocytes were isolated with hyaluronidase (Sigma-Aldrich; Cat# 37326-33-3) and repeatedly pipetting.

For the collection of MII-stage oocytes, 4–5 weeks old females were injected with 10U of PMSG and after 46–48 h with 10U of human chorionic gonadotropin (hCG) (Ningbo Second Hormone Factory). Cumulus-enclosed oocytes were isolated from the oviduct 14 h after hCG stimulation. MII-stage oocytes with a first polar body were separated from the cumulus cells with hyaluronidase and repeatedly pipetting in M2 media.

### NAT10 Knockdown by Electroporation and Oocyte *in vitro* Maturation

Three specific small interfering RNAs (siRNAs) specifically targeting mouse NAT10 transcripts were synthesized by GenePharma Company (Shanghai, China). The sequences were listed in Supplementary Table 1. Electroporation was employed to deliver siRNAs into GV-stage oocytes using Pulse Generator CUY21EDIT II (BEX Co., Ltd., Tokyo, Japan) according to the manufacturer’s instructions. Briefly, denuded GV-stage oocytes were incubated in prewarmed Tyrode’s solution (Leagene, Beijing, China; Cat# CZ0060) for 10s to weaken the zona pellucida. The siRNAs were diluted to 400nM in OPTI-MEM (Gibco, California, USA; Cat# 31985-062). GV-stage oocytes were transferred to the siRNA-containing Opti-MEM medium. Then oocytes and medium (total volume of 5 μl) were added into a flat electrode chamber and arranged linearly between two paralleled electrodes. The electroporation parameters were 30 volts in amplitude, 1 ms pulse width and 4 pulses at intervals of 50 ms. After electroporation, the oocytes were immediately washed three times and placed in Opti-MEM medium. Thirty minutes later, oocytes were transferred into IVM medium supplemented with 50 μM 3-isobutyl-1-methyl-xanthine (IBMX) (MCE, Shanghai, China; Cat# HY-12318). The basic IVM medium was TCM-199 (Gibco; Cat# 31100035) supplemented with 0.2 mM sodium pyruvate and 10% fetal bovine serum (FBS). The oocytes were cultured in IBMX-containing medium for 24 h in a humidified atmosphere of 5% CO_2_/95% air at 37°C, to keep them arrested at GV stage and facilitate the degradation of NAT10 mRNA. Oocytes were then washed and incubated in IBMX-free IVM medium for another 14 h, followed by observation of oocyte morphology under light microscope and calculation of maturation rates.

### Immunofluorescence Staining of Oocytes

Denuded oocytes were fixed and permeabilized using 1% paraformaldehyde, 0.2% Triton X-100 in phosphate buffered saline (PBS) for 1 h at room temperature, and then blocked in 3% bovine serum albumin (BSA) in PBS for 1 h. Incubation with first antibodies against ac4C (1:200, abcam, Cambridge, UK; Cat# ab252215) or NAT10 (1:200, ProteinTech, Wuhan, China; Cat# 13365-1) overnight was performed at 4°C, followed by three washes (5min each time) with 0.3% BSA. Incubation with cy3-conjugated secondary antibody (1:500, Earthox, CA, United States; E031620) was performed for 1 h at room temperature out of light, also followed by three washes with 0.3% BSA. Images were taken under an inverted fluorescence microscope IX73 (Olympus, Tokyo, Japan).

### Immunoblot Assay of Oocytes

A total of 200 oocytes were collected in 20 μl RIPA buffer (CWBIO, Beijing, China; Cat# CW2333S) and incubated at 95°C for 5 min in 2 × SDS loading buffer (GenStar, Beijing, China; Cat# E151-10). For dot blot, protein extracts were directly loaded to polyvinylidene fluoride membranes (Genstar; Cat# E195-201). For western blot, lysates were separated on 10% gels via SDS-PAGE and then transferred to the membranes. Thereafter, membranes were blocked with 5% skimmed milk for 1 h and incubated at 4°C overnight with the primary antibody against TBL3 (1:1,000, ABclonal, Wuhan, China; Cat# A10043). Detection of actin with antibody (Sigma-Aldrich; Cat# A2066) was used as an internal control. After three washes with PBST (0.2% Triton X-100 in PBS) and incubation with HRP-conjugated secondary anti-rabbit IgG antibody (1:2,000, Earthox; Cat# E030120-02), the membrane was scanned with a BIO-RAD ChemiDoc^TM^ Imaging System.

### Cell Lines and Transfection

Human embryonic kidney HEK293T cells and mouse Leydig TM3 cells (FuHeng Biology, Shanghai, China; Cat# FH0244 and FH0337, respectively) were maintained in high-glucose DMEM medium (Gibco; Cat# C11960500BT) supplemented with 10% FBS and 1% penicillin-streptomycin at 37°C and 5% CO_2_ in a humidified atmosphere. HEK293T cells were transfected with NAT10-overexpressing plasmids (GeneCopeia, Guangzhou, China; Cat# EX-I5674-M11) with Lipofectamine^TM^ 3000 Transfection Reagent (Thermo Fisher Scientific, MA, United States; Cat# L3000001) according to manufacturer’s instructions. To downregulate NAT10 expression in TM3 cells, siRNAs were introduced into cells using Lipofectamine^TM^ 3000 Transfection Reagent.

### RNA Extraction and Quantitative Real-Time PCR

Total RNA was extracted from cultured HEK293T cells using RNeasy Micro Kit (Qiagen, MD, USA; Cat# 74004) according to manufacturer’s instructions. First-strand complementary DNAs (cDNAs) were synthesized by reverse transcription using HiScript III RT SuperMix for qPCR (Vazyme, Nanjing, China, Cat# R323-01). Quantitative PCR (qPCR) was carried out using 2x RealStar Green Power Mixture (Genstar; Cat# A311-101) on a Roche LightCycler 480 II (Roche Diagnostics, Mannheim, Germany). The expression of β-actin was used as an endogenous control. The primers used in this study were listed as follows:

NAT10: forward: TTGGCTGGCAGCATTTTGGA,
  reverse: GGCTGACTTGGCTACGTTCC;
-actin: forward: GTGACGTTGACATCCGTAAAGA,
  reverse: GCCGGACTCATCGTACTCC.

### Dot Blot of RNA ac4C Modification

Dot blots were performed using a rabbit monoclonal anti-ac4C antibody (abcam; Cat# ab252215) as previously described (Sinclair et al., 2017). Briefly, indicated amounts of extracted RNA or synthetic RNA probes were denatured at 65°C for 5 min, followed by immediately chilling on ice for 2 min and spotted onto Hybond-N + membranes (GE Healthcare, Shanghai, China; Cat# RPN303B). After UV crosslinking, the membranes were blocked with 5% skimmed milk in PBST for 1 h at room temperature, and incubated overnight with anti-ac4C antibody in 5% BSA (1:1,000) at 4°C. Membranes were next washed three times with 0.1% PBST, incubated with HRP-conjugated secondary anti-rabbit IgG antibody (1:2,000, Earthox; E030120-02) in 5% skimmed milk for 1 h at room temperature, and scanned with a ChemiDoc^TM^ Imaging System (BIO-RAD, CA, United States). Quantification of blot intensity was performed using ImageJ software (version 1.8.0, NIH, MD).

### RNA-Seq and Data Analysis

Total RNA was extracted from NAT10-overexpressed or control HEK293T cells. High-throughput sequencing was performed at Magigen (Guangzhou, China). Briefly, purified RNA was first assessed for quality. After isolation and fragmentation of mRNA, cDNAs were synthesized. The fragments were then subjected to end-repair, 3′ adenylation and adaptors ligation. Suitable fragments were amplified by PCR for 13 cycles. The PCR products were cleaned up using beads, validated using an Qsep100 (Bioptic, Taiwan, China), and quantified by Qubit3.0 Fluorometer (Invitrogen, CA, United States). Then libraries with different indices were multiplexed and loaded on an Illumina HiSeq instrument according to manufacturer’s instructions (Illumina, CA, United States). Sequencing was carried out using a 2 × 150 bp paired-end (PE) configuration. All reads were mapped to human genome version hg19 by Hisat2 (v2.0.1) with default settings and reads count was converted to CPM (counts per million). Differential expression of genes was analyzed in edgeR. *P*-value and fold change (FC) of each gene were calculated. Genes showing altered expression with *p* < 0.05 and | logFC| > 1 was were considered as differentially expressed genes (DEGs). Using DAVID,^1^ the gene ontology (GO) enrichment including biological processes (BP), cellular components (CC) and molecular function (MF) were conducted, and Kyoto Encyclopedia of Genes and Genomes (KEGG) pathway analysis was performed for DEGs. Bubble plots and bar plots were plotted by http://www.bioinformatics.com.cn, an online platform for data analysis and visualization.

### NAT10 RNA Immunoprecipitation Sequencing (RIP-Seq) and Data Processing

NAT10-overexpressed HEK293T cells with 80% confluency in 75 cm^2^ culture flasks were rinsed with cold PBS, placed on ice and detached mechanically using a cell scraper. Cells were centrifuged at 2,000 rpm for 8 min at 4°C and resuspended in 1 ml lysis buffer (50 mM HEPES [pH 7.5], 150 mM NaCl, 1mM DTT, 0.005% Triton X-100) supplemented with 0.5% NP40 and 1% protease inhibitor cocktail (APExBIO, TX, United States; Cat# K1007). Lysates were treated with 2 U/ml DNase I (Thermo Fisher Scientific; Cat# 18047019) at 37° for 5 min and immediately put on ice followed by clearing at 13,000 rpm for 5 min. Hundred microliter of cell lysates was reserved as input. Per each immunoprecipitation, 2.5μg polyclonal anti-NAT10 antibody (ProteinTech; Cat#:13365-1) or 2.5μg rabbit IgG control (FineTest, Wuhan, China; Cat# FNSA-0106) was conjugated to Protein A/G Magnetic Beads (MCE; Cat# HY-K0202) by incubation for 2 h at room temperature, followed by three washes and incubation with cell lysates for 4 h at 4°, supplemented with 36 μl 0.25M EDTA and 1 μl RNase inhibitor (APExBIO; Cat# K1046). After washing with lysis buffer for three times, the conjugated beads were incubated with 150 μl proteinase K buffer (117 μl lysis buffer, 15 μl 10% SDS, 18 μl 10 mg/ml proteinase K) for 30 min at 55°, with constant rotation. RNA was next extracted by phenol-chloroform-isoamyl alcohol mixture [pH < 5.0] and ethanol precipitation using 3M sodium acetate [pH 5.5], and analyzed by RNA-seq as described above. The RIP targets were defined as genes with CPM ≥ 1, IP/input ≥ 2, and *p* < 0.05, as described previously in the literature (Huang et al., 2018).

### *In vitro* Transcription and Purification of ac4C-Containing RNA Probes

RNA probes containing ac4C were synthesized as previously described (Sinclair et al., 2017). Briefly, DNA templates of the probes were *in vitro* transcribed using the T7 High Yield RNA Synthesis Kit (APExBIO; Cat# K1047) according to the manufacturer’s instructions. For acetylated transcripts, ac4CTP (MCE, Cat# HY-111815A) replaced CTP in the reaction mix. For both acetylated and unacetylated probes, 25% of UTP was replaced with biotin-16-UTP (Lucigen, WI, United States; Cat# BU6105H). Synthesized probes were purified by VAHTS RNA Clean Beads (Vazyme; Cat# N412) according to the manufacturer’s instructions. Specifically, the volume of RNA Clean Beads was 2.5-fold of the RNA solution considering the short length of RNA probes. The sequences of probes used in this study were listed in Supplementary Table 2.

### Prediction of ac4C Binding Proteins

Potential ac4C binding proteins were predicted by the online algorithms catRAPID^2^ (Agostini et al., 2013). We processed a catRAPID analysis and obtained the predicated binding proteins of ac4C sites. Briefly, the motifs enriched within ac4C peaks were obtained from the literature (Arango et al., 2018). We arranged the motifs in a tandem array and subjected this sequence to catRAPID omics to investigate its interactions with RNA-binding proteins (RBPs).

### Biotin-RNA Pulldown Assay

HEK293T cells were harvested at 80% confluence, washed with PBS and lysed in lysis buffer (50 mM HEPES [pH 7.5], 150 mM NaCl, 1 mM DTT, 0.005% Triton X-100) containing 1% protease inhibitor cocktail. Lysates were separated from insoluble cell debris by centrifugation (18,000 g for 10 min at 4°C), supplemented with 1 μg/μl RNase inhibitor and incubated with 100 μg yeast tRNA (Solarbio, Beijing, China; Cat# T8630) for 1 h at 4°C. Three microgram biotinylated RNA probes were denatured at 90°C for 2 min and immediately put on ice for at least 2 min. The RNA probes were mixed with Annealing Buffer for RNA oligos (5X) (Beyotime, Shanghai, China; Cat# R0051) and incubated for 20 min at room temperature, followed by incubation with pretreated cell lysates with constant mixing for 1 h. For each reaction, 50 μl streptavidin magnetic beads (Invitrogen; Cat# 65601) were pretreated with 100 μg yeast tRNA for 20 min and incubated with the above-mentioned cell lysates containing RNA-protein complexes for 30 min. After extensive washing, magnetic beads conjugated with RNA–protein complexes were resuspended in RIPA lysis buffer (Genstar; Cat# E121-01). The proteins interacting with the RNA probes were subsequently detected by liquid chromatography-mass spectroscopy/mass spectroscopy (LC-MS/MS) or western blotting analysis. The label-free mass spectrometry analysis and data processing were performed by GeneCreate Biological Engineering Co., Ltd. (Wuhan, China).

### Statistical Analysis

Statistical methods were adopted as appropriate using SPSS19.0 (SPSS Inc., IL, United States) and GraphPad Prism7 (GraphPad Software, CA, United States). Measurement data are presented as mean (SD) and Student’s *t*-test (two-tails) was used for comparison between two groups. Differences among more than two groups were estimated using one-way ANOVA. Counting data were expressed as n (%) and comparison was evaluated by Chi-square test. Results were considered statistically significant when *p* < 0.05.

## Results

### ac4C and NAT10 Were Downregulated During Oocyte Maturation

**FIGURE 1:**
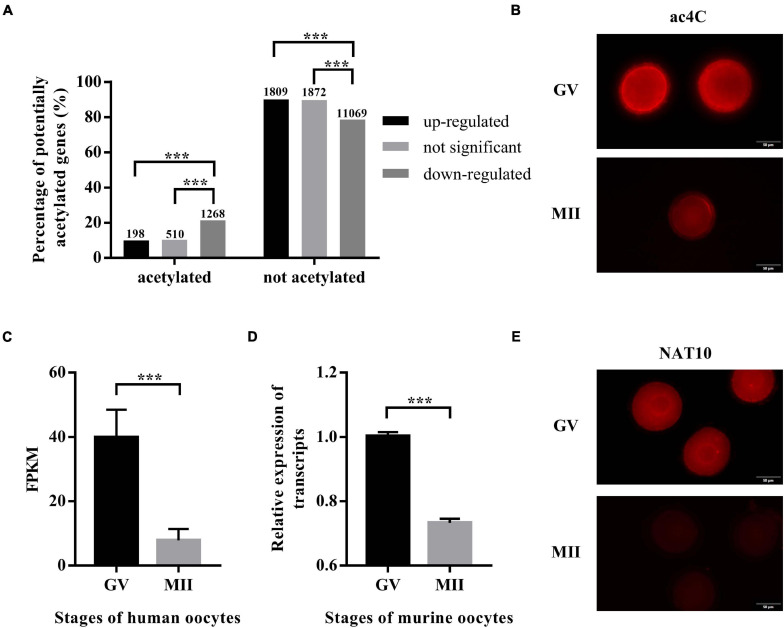
ac4C and NAT10 levels at defined stages of oocyte meiotic progression. **(A)** Distribution of potential ac4C sites in differentially expressed genes between GV and MII oocytes. The numbers of genes in each category were shown above the bars. These data were obtained from GEO (GSE102113, GSE95477). **(B)** RNA ac4C modification during mouse oocyte maturation. Exposure time: GV, 300 ms; MII, 1.9 s. Scale bar=50 μm. **(C)** Transcriptional levels of NAT10 in human oocytes, logFC = –2.35. Data were obtained from GEO (GSE95477). **(D)** Transcriptional levels of NAT10 in mouse oocytes, logFC = –2.31. Data were obtained from GEO (GSE5668). **(E)** NAT10 expression during mouse oocyte maturation. Exposure time: GV, 93 ms; MII, 200 ms. Scale bar=50 μm. GV, germinal vesicle. MII, metaphase II. FC, fold change. FPKM, fragments per kilobase per million. The immunostaining was repeated in at least three independent experiments. ***Significant difference at *p* < 0.001.

The expression profiles of human oocytes (GSE95477) were retrieved from the Gene Expression Omnibus (GEO).^3^ Data of GV-stage oocytes and MII-stage oocytes maturated *in vitro* were included in this study. Differential expression analysis identified 2,007 and 2,382 genes that were up- and down-regulated during IVM, respectively, while 12,337 genes remained constant. To explore whether ac4C modification plays a role in oocyte maturation, transcripts with potential ac4C sites were obtained from a previous study (Arango et al., 2018), and we calculated the percentages of potentially acetylated transcripts in the up-regulated, down-regulated, and unchanged genes. Interestingly, transcripts with potential ac4C sites were enriched in down-regulated genes (Figure 1A). Next, immunostaining performed using an antibody against ac4C modification on mouse oocytes revealed a significantly decreasing trend from GV to MII oocytes (Figure 1B).

Since NAT10 is the only known RNA acetyltransferase responsible for ac4C formation in human (Arango et al., 2018; Dominissini and Rechavi, 2018), we further investigated its expression in oocytes at different stages. Data on NAT10 expression were extracted from publicly available sequencing profiles on GEO (GSE95477, GSE5668). Markedly decreased expression of NAT10 during oocyte meiotic progression was observed in both human and murine oocytes (Figures 1C,D). Similarly, immunostaining revealed a marked decrease at protein level, consistent with the transcript level (Figure 1E). Taken together, we confirmed the down-regulated expression of both ac4C and NAT10 from GV to MII oocytes, which suggested a possible role of NAT10-mediated RNA ac4C modification in modulating oocyte maturation.

### NAT10 Depletion Retarded Meiotic Progression in Mouse Oocytes

**FIGURE 2:**
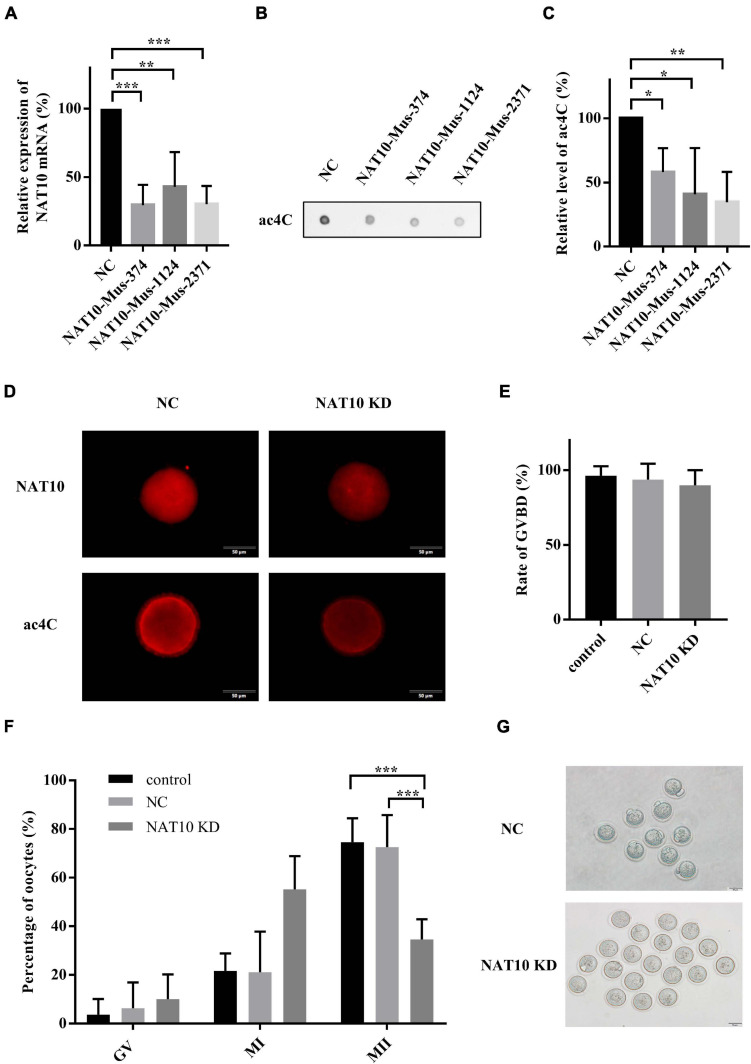
NAT10 knockdown retarded meiotic progression in mouse oocytes. **(A)** Relative expression of NAT10 mRNA with NAT10 knockdown in TM3 cells. **(B,C)** ac4C was downregulated by NAT10 depletion in TM3 cells. **(D)** Representative images of NAT10 and ac4C immunostaining in NC and NAT10 KD oocytes. Scale bar=50 μm. **(E)** Quantitative analysis of the GVBD rates in control, NC and NAT10-knockdown oocytes. **(F)** Percentages of oocytes at different stages after *in vitro* culture. **(G)** Phase-contrast images of NC and NAT10 KD oocytes. Scale bar=50 μm. The data were presented as the mean ± SD from at least three independent experiments. NC, negative control siRNA. NAT10 KD, NAT10 knockdown by siRNA (NAT10-Mus-2371). Control, oocytes without transfection. GVBD, germinal vesicle breakdown. *Significant difference at *p* < 0.05, ** at *p* < 0.01, and *** at *p* < 0.001.

To reduce NAT10-mediated ac4C modification, three NAT10-specific siRNAs were synthesized (Supplementary Table 1). Effective knockdown of NAT10 mRNA was confirmed by RT-qPCR analysis in a murine somatic cell line TM3 (Figure 2A). Dot blot showed significant decrease of ac4C modification with NAT10 depletion (Figures 2B,C). Among three siRNAs specific to mouse NAT10, NAT10-Mus-2371 with significant reduction of both NAT10 and ac4C was selected for subsequent experiments. Fully grown GV-stage oocytes were transfected with NAT10 siRNA by electroporation and incubated in IBMX-supplemented medium for 24 h to facilitate the degradation of NAT10 mRNA. NAT10 knockdown and ac4C reduction in GV oocytes were confirmed by immunofluorescence staining (Figure 2D).

After 24 h in IBMX-supplemented medium, the oocytes transfected with siRNAs were continuously cultured in IBMX-free medium for another 14 h. The rate of germinal vesicle breakdown (GVBD) was not affected by the transfection of NAT10 siRNA. The average rates of GVBD were 89.9, 93.7, and 96.3% in control oocytes without transfection, negative control siRNA and NAT10 knockdown groups, respectively (*p* = 0.6531) (Figure 2E). The rate of first body extrusion (MII oocytes), however, was significantly decreased with NAT10 knockdown (74.6% of control without transfection; 72.6% of negative control siRNA; 34.6% of NAT10 knockdown; *p* < 0.001) (Figures 2F,G). Taken together, NAT10 depletion led to a disturbance of meiosis, possibly via reduction of RNA ac4C modification. These results indicated that NAT10-mediated ac4C modification plays a critical role in modulating oocyte meiotic progression.

### Functional Enrichment Analysis of NAT10-Binding Transcripts

**FIGURE 3:**
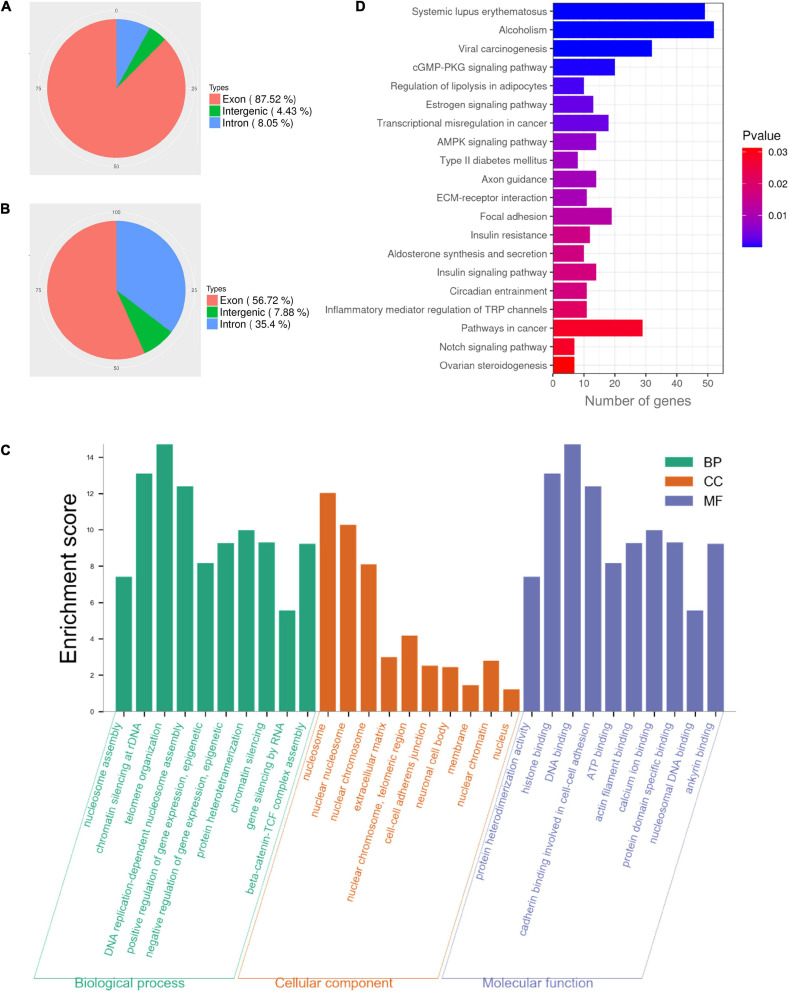
Functional enrichment analysis of NAT10-binding transcripts in HEK293T cells. **(A)** Distribution of reads from input. **(B)** Distribution of NAT10-binding reads. **(C)** GO enrichment analysis of RIP-binding transcripts. **(D)** KEGG analysis of NAT10-RIP transcripts. RIP, RNA immunoprecipitation. GO, Gene Ontology. BP, Biological Processes. MF, Molecular Functions. CC, Cellular Components. KEGG, Kyoto Encyclopedia of Genes and Genomes.

Our results demonstrated that NAT10 knockdown resulted in decreased rate of first polar body extrusion in mouse oocytes. To clarify the regulated genes, we performed NAT10 RIP-seq to identify NAT10-targeted transcripts. The procedure of RIP requires a large number of cells and cannot be performed with the limited amount of RNA that can be obtained from oocytes. Thus, we used HEK293T cells instead. The genes with CPM ≥ 1, IP/input ≥ 2, and p-value < 0.05 were considered as RIP targets, and a total of 1,296 genes were identified. As shown in Figures 3A,B. 87.52% of total reads from the input were mapped to exons in the genome, while 56.72% of reads from the immunoprecipitated sample were mapped to exons.

In previous literature, proliferation kinetics was reduced in NAT10 knockout Hela cells. Functional annotation revealed that genes associated with cell adhesion, regulation of apoptosis, small GTPase, extracellular matrix and cell proliferation were dysregulated with NAT10 depletion (Arango et al., 2018). To understand the functions of the NAT10-binding transcripts identified in our study, all 1,296 identified genes were subjected to GO and KEGG enrichment analyses. The results revealed that NAT10-binding genes were significantly enriched in the biological processes associated with nucleosome assembly, chromatin silencing at rDNA, telomere organization, DNA replication-dependent nucleosome assembly, and positive epigenetic regulation of gene expression. As for cellular components, the targeted genes were enriched in components of nucleosome, nuclear chromosome, extracellular matrix, cell-cell adherents junction, and nuclear chromatin. According to molecular functions, these genes were typically enriched in protein heterodimerization activity, histone binding, DNA binding, cadherin binding involved in cell-cell adhesion, and ATP binding (Figure 3C). Analysis of KEGG pathway showed that NAT10-binding genes were enriched in cGMP-PKG signaling pathway, estrogen signaling pathway, transcriptional misregulation, and AMPK signaling pathway (Figure 3D).

### Identification of Genes Modulated by NAT10-Mediated ac4C Modification

**FIGURE 4:**
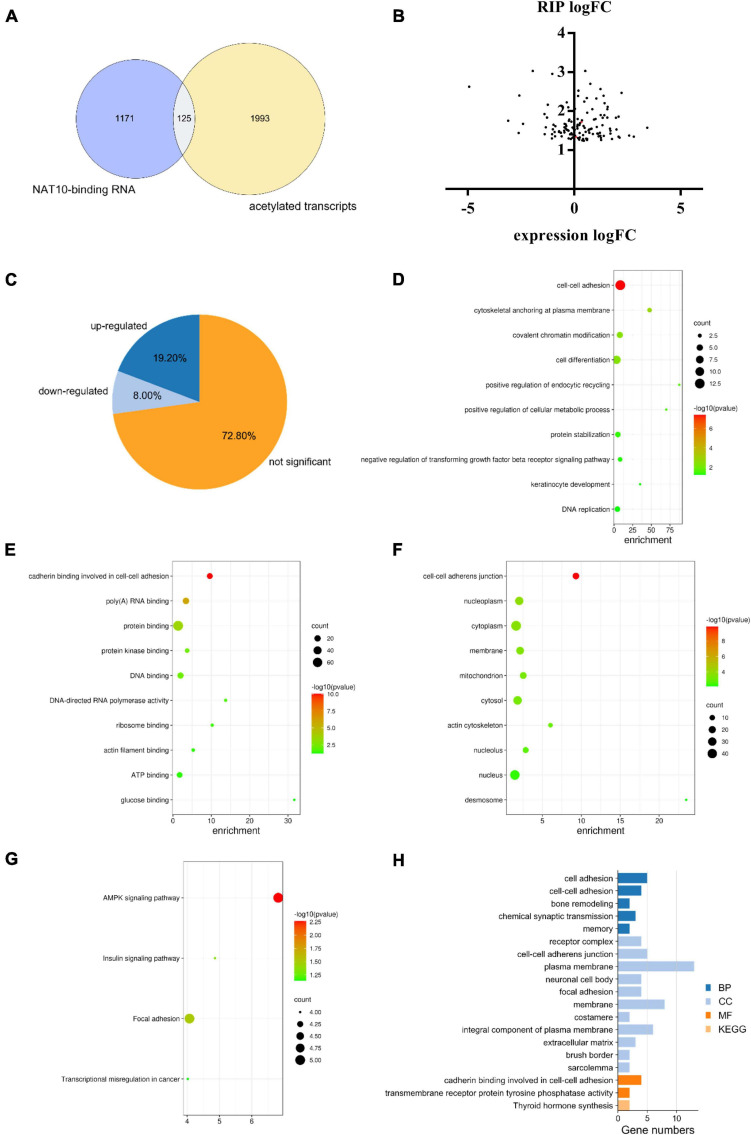
Combined analysis of RIP-seq, transcriptome and acetylated transcripts. **(A)** Overlap of ac4C transcripts and NAT10-binding RNA identified by RIP-seq. ac4C transcripts were obtained from GEO database (GSE102113), and RIP-seq was performed in this study using HEK293T cells. **(B)** Expression of NAT10-binding genes in NAT10-overexpressed HEK293T cells. **(C)** Proportions of up-regulated, down-regulated and not significant genes in the 125 NAT10-binding genes with potential ac4C sites. **(D–F)** GO enrichment on the subset of down-regulated and not significant genes from **(C)**, including **(D)** BP, **(E)** CC, and **(F)** MF. **(G)** KEGG pathway analysis on the down-regulated and not significant genes from **(C)**. **(H)** GO enrichment and KEGG pathway analysis on the subset of up-regulated genes from **(C)**. RIP-seq, RNA immunoprecipitation and high-through sequencing. GO, Gene Ontology. BP, Biological Processes. MF, Molecular Functions. CC, Cellular Components. KEGG, Kyoto Encyclopedia of Genes and Genomes.

A total of 2,118 transcripts with potential ac4C modification were obtained from a previous study (Arango et al., 2018). Among the NAT10-binding genes identified in our study, there were 125 transcripts with potential ac4C sites (Figure 4A), which can potentially be modulated by NAT10-mediated ac4C modification. Since the known functions of ac4C in mRNA were to stabilize RNA and promote translation efficiency (Dominissini and Rechavi, 2018), we further investigated the transcript levels of these 125 genes with NAT10 overexpression (Figure 4B). We presumed that the stability of up-regulated genes was promoted by ac4C modification, resulting in increased transcript levels, while ac4C regulates the other genes by promoting translation efficiency without affecting transcript levels. As shown in Figure 4C and Supplementary Table 3, 24 genes were up-regulated with NAT10 overexpression, while 101 genes were down-regulated or not significantly changed. Functional annotation showed that the 101 down-regulated or not significant genes were enriched in biological functions as cell-cell adhesion, cytoskeletal anchoring at plasma membrane, and covalent chromatin modification (Figures 4D–G). Pathway analysis revealed that the up-regulated genes were associated with cadherin binding involved in cell-cell adhesion and transmembrane receptor protein tyrosine phosphatase activity (Figure 4H).

A long-standing hypothesis indicates that an intracellular second messenger, cyclic adenosine monophosphate (cAMP), plays a critical role in maintaining meiotic arrest of oocytes (Pan and Li, 2019). Adenylyl cyclases (ADCYs) are well-characterized enzymes that catalyze the cyclization of adenosine triphosphate (ATP) into cAMP, thus keeping a high level of intracellular cAMP and maintaining meiotic arrest (Zhang and Xia, 2012; Khannpnavar et al., 2020). Among the nine transmembrane-bound ADCY genes (ADCY 1–9) found in human genome, the functions of ADCY1, ADCY3 and ADCY9 have been described in oocytes (Pan and Li, 2019). Our data demonstrated that ADCY3, the major ADCY in mouse oocyte (DiLuigi et al., 2008), is an NAT10-binding transcript with potential ac4C-modified sites, and its expression was up-regulated with NAT10 overexpression. Publicly available expression profiles on human oocytes (GSE95477) showed that ADCY3 was down-regulated during maturation (logFC = −2.09, *P* < 0.001), as included in the category of acetylated down-regulated transcripts in Figure 1A. Our team is working on single cell sequencing of oocytes (full data not shown) and the data also showed that NAT10 knockdown led to significantly decreased expression of ADCY3 (logFC = −2.36, P = 0.043). But the results reported from some previous literature showed that the effects of ADCY3 deficiency on oocyte meiosis resumption were conflicting, possibly due to the crosstalk and interaction of the signaling pathways (DiLuigi et al., 2008; Xiong et al., 2017). Still, dysregulation of ADCY3 mediated by ac4C modification could be a potential mechanism of retarded oocyte maturation *in vitro* with NAT10 knockdown.

In addition, we searched in PubMed^4^ using “oocyte” and the genes as keywords, in order to identify genes with reported functions in regulating oocyte maturation. As shown in Supplementary Table 4, among the 125 transcripts with potential ac4C sites, 28 genes were reported to play a regulatory role in oocyte meiosis. This implicates potential mechanisms via which NAT10-mediated ac4C modification influences oocyte maturation.

### Identification of Potential ac4C-Binding Proteins

As a newly identified mRNA modification, the regulation of ac4C remains largely unexplored. Although a few studies have confirmed that NAT10 and its homologs are the source of ac4C production (writer) (Ito et al., 2014; Sharma et al., 2015; Dominissini and Rechavi, 2018), RBPs that specifically binds to ac4C modification and mediate downstream cellular activities (readers) have not been reported yet. To predict protein-RNA interactions, we first processed a catRAPID analysis with ac4C motifs and obtained a serial of potential ac4C-binding proteins. The predicted RBPs are ranked based on their scores from high to low (Supplementary Table 5).

**FIGURE 5:**
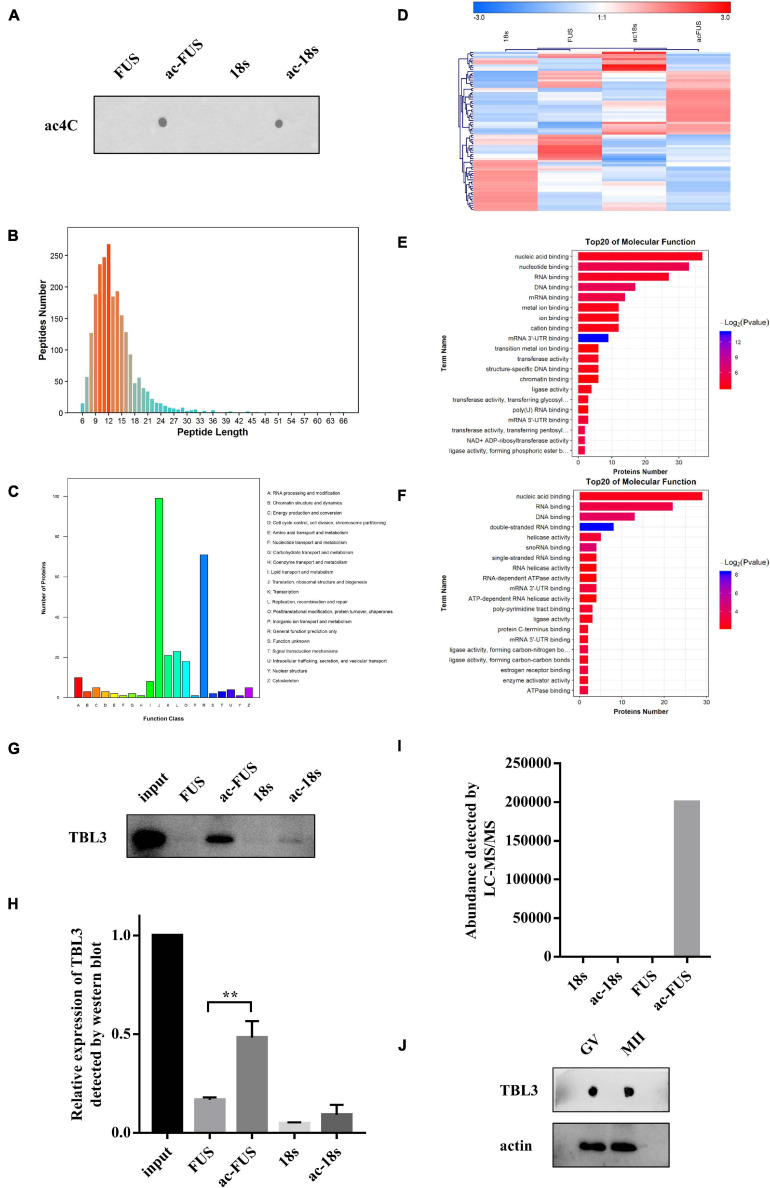
Identification of potential ac4C-binding proteins using HEK293T cells. **(A)** ac4C dot blot of synthesized acetylated and non-acetylated RNA probes. **(B)** Distribution of peptide length identified by LC-MS/MS. **(C)** Functional classification of identified proteins according to COG classification. **(D)** Hierarchical clustering of differentially expressed proteins. **(E)** GO enrichment of molecular function, FUS vs. ac-FUS. **(F)** GO enrichment of molecular function, 18s vs. ac-18s. **(G,H)** Expression of TBL3 in input and RNA pulldown samples. The data are presented as the mean ± SD from three independent experiments. **Significant difference at *p* < 0.01. **(I)** TBL3 abundance identified by mass spectrometry analysis. **(J)** Immunoblot of TBL3 in mouse oocytes. LC-MS/MS, liquid chromatography-mass spectroscopy/mass spectroscopy. COG, Cluster of Orthologous Groups of proteins. GO, Gene Ontology. FUS, synthetic RNA probe consisting of the acetylated fragment of FUS gene. ac-FUS, ac4C-modified FUS probe. 18s, synthetic RNA probe consisting of the acetylated fragment of 18s rRNA. ac-18s, ac4C-modified 18s probe. GV, germinal vesicle. MII, metaphase II.

Meanwhile, we synthesized acetylated and non-acetylated RNA probes by *in vitro* transcription (Figure 5A). The sequences of the probes were segments of FUS and 18s rRNA, which contain ac4C sites as reported in literature (Ito et al., 2014; Arango et al., 2018). A biotin-RNA pulldown assay and mass spectrometry were performed with HEK 293T cell lysates. As shown in Table 1, 1,948 peptides and 374 proteins were identified. Distribution of peptide length detected in all samples was shown in Figure 5B. Functional classification of all identified proteins was carried out using the COG (Clusters of Orthologous Groups of Proteins) database (Tatusov et al., 2003). A majority of identified RBPs were enriched in functions associated with translation, ribosomal structure and biogenesis, transcription, posttranscriptional modification, protein turnover, chaperones, RNA processing and modification (Figure 5C). As for differential expression analysis, 55 proteins were significant different between 18s and ac-18s, and 73 RBPs showed different abundance between FUS and ac-FUS (Table 2). The heatmap demonstrated several clusters of differential abundance (Figure 5D). Functional annotation revealed that the differential proteins are enriched in molecular functions associate with nucleic acid binding, nucleotide binding and RNA binding (Figures 5E,F).

**TABLE 1:** Number of proteins identified by mass spectrometry using HEK293T cells.

Type	Spectrum	Peptides	Proteins
18s	7,136	1,201	229
FUS	6,164	1,158	248
ac-18s	7,714	1,235	259
ac-FUS	6,904	1,150	278
Combined	27,918	1,948	374

**TABLE 2:** Differentially expressed proteins identified by mass spectrometry using HEK293T cells.

Type	ac-18s/18s	ac-FUS/FUS
Quant Num.	202	205
Sig. diff Num.	55	73
Sig. up num.	23	36
Sig. down num.	32	37

*Quant Num., number of groups of quantified proteins. Sig. diff Num., number of differentially expressed proteins. Sig. Up Num., number of significantly up-regulated proteins. Sig. Down Num., number of significantly down-regulated proteins.*

### TBL3 Specifically Bound to Acetylated RNA Probes

We further combined the results of bioinformatic prediction and mass spectrometry (Table 3). Among the top 10 RBPs predicted by catRAPID, TBL3, HNRDL, and PTBP1 were detected in mass spectrometry. But only TBL3 specifically bound to ac4C probes using HEK293T cells (Figure 5I). Meanwhile, TBL3 showed the highest score, discriminative power and interaction strength as predicted by the algorithm (Supplementary Table 5). To further confirm the interaction, we performed RNA pulldown and western blotting. The results demonstrated that TBL3 specifically bound to ac-FUS, consistent with the result of mass spectrometry (Figures 5G,H). As for the expression of TBL3 in oocytes, online expression profiles (GSE95477) showed that TBL3 mRNA was detected in human oocytes and was down-regulated during meiotic maturation (logFC = −2.22, *P* = 0.00052). Immunoblot assays demonstrated that TBL3 was expressed in mouse oocytes, but no significant difference in expression was observed between GV and MII oocytes (Figure 5J). Collectively, our data suggest that TBL3 is a potential ac4C reader, which may mediate the downstream biological functions of ac4C modification.

**TABLE 3:** Top 10 RNA-binding proteins predicted by catRAPID and their abundance in mass spectrometry.

Rank	Protein	Description*	Abundance detected by mass spectrometry			
			18s	ac-18s	FUS	ac-FUS
1	TBL3	Transducin beta-like protein 3	–	–	–	200,111
2	MGN2	Protein mago nashi homolog 2	–	–	–	–
3	MGN	Protein mago nashi homolog	–	–	–	–
4	PIWL2	Piwi-like protein 2	–	–	–	–
5	LRRF2	Leucine-rich repeat flightless-interacting protein 2	–	–	–	–
6	MSH4	MutS protein homolog 4	–	–	–	–
7	EXOS6	Exosome complex component MTR3	–	–	–	–
8	POP1	Ribonucleases P/MRP protein subunit POP1	–	–	–	–
9	SF3B2	Splicing factor 3B subunit 2	–	–	–	–
10	HNRDL	Heterogeneous nuclear ribonucleoprotein D-like	2,314,101	1,736,563	572,541	837,440
10	PTBP1	Polypyrimidine tract-binding protein 1	723,724	466,609	321,505	–

*Annotation was made according to UniProt database (https://www.uniprot.org/).

## Discussion

In fully grown mammalian GV-stage oocytes, transcription ceases and maternal transcripts accumulate, which support subsequent processes including meiotic maturation, fertilization and early embryo development (Walls and Hart, 2018; Esencan et al., 2019; Toralova et al., 2020). With oocyte meiosis resuming triggered by hormones, approximately 20% of total maternal transcriptome were selectively degraded (Ivanova et al., 2017), while a large number of specific transcripts were translationally activated by MAPK pathway (Brachova et al., 2021). mRNA decay and translational regulation are fundamental events in oocyte meiotic maturation.

Accumulating evidence have shown that epigenetic modifications of mRNA are crucial for RNA biology (Yue et al., 2015; Kumar and Mohapatra, 2021), including translation (Meyer et al., 2015; Huang et al., 2018), splicing (Xiao et al., 2016), transport (Roundtree et al., 2017) and degradation (Wang et al., 2014; Huang et al., 2018). Two m6A readers, YTHDF2 and YTHDC1, have been reported to play an important role in mouse oocyte maturation. During meiotic progress, YTHDF2 binds and destabilizes m6A-modified transcripts (Ivanova et al., 2017), and YTHDC1 is required for alternative splicing in oocytes (Kasowitz et al., 2018). Another study demonstrated that deficiency of inosine RNA modification in GV-stage mouse oocytes impaired mRNA decay (Brachova et al., 2021). The newly described ac4C is the only known acetylation within mRNAs. It is widely distributed across the transcriptome and has an intrinsic role in promoting the stability and translational efficiency of mRNAs. Considering the large number of ac4C-targeted transcripts, it is believed that ac4C is an important component of epigenetic modulation (Arango et al., 2018; Dominissini and Rechavi, 2018). Although previous work has established that ac4C can impact mRNA stability and translation, the relationship of ac4C and oocyte maturation has not yet been demonstrated.

In this study, we described the down-regulated expression of ac4C during mouse oocyte maturation. We also assessed the expression of NAT10, the only known RNA acetyltransferase in mammals, and observed downward trends at both mRNA and protein levels. These results implicated that NAT10-mediated ac4C is potentially involved in the post-transcriptional modulation of oocyte maturation. We further reduced ac4C modification in GV-stage oocytes by NAT10 knockdown. The maturation rate of IVM significantly decreased, implying that NAT10-mediated ac4C is crucial for oocyte meiosis. We also performed NAT10 RIP-seq and high-throughput sequencing to determine the regulated genes. Combined analysis with acetylated transcripts showed a series of mRNAs that were potentially modulated by NAT10-mediated ac4C. The dysregulated genes were enriched in biological processes associated with oocyte maturation, including nucleosome assembly, chromatin silencing, chromatin modification and cytoskeletal anchoring. Although ac4C can promote mRNA stability and translation, we cannot clarify the exact impact that ac4C exerted on a certain transcript due to technical limitations.

Specifically, we identified ADCY3 as a transcript modulated by NAT10-mediated ac4C. ADCY3 is an important source of cAMP in GV oocytes, which maintains meiotic arrest (Khannpnavar et al., 2020). During oocyte maturation, ADCY3 is down-regulated, consistent with the trends of NAT10 and ac4C. Since ac4C promotes mRNA stability, we hypothesize that decline in NAT10 expression and ac4C modification results in degradation of ADCY3, leading to a decreased level of intracellular cAMP, ultimately contributing to stimulation of meiotic resumption. But with NAT10 knockdown, ADCY3 was abnormally reduced, resulting in retarded maturation. Previous reported data on the effects of ADCY3 deficiency were also inconsistent (DiLuigi et al., 2008; Xiong et al., 2017). Although accumulation of cAMP in oocytes is critical in maintaining meiotic arrest (Pan and Li, 2019), reduced cAMP level alone may not be sufficient to trigger meiosis. Instead, abnormally reduced ADCY3 and cAMP may even impair meiosis due to crosstalk and interaction of various signaling pathways. That explains the retarded maturation observed in our study.

Interestingly, ac4C modification is known to exist within mRNAs, but the results of NAT10 RIP-seq revealed that NAT10-binding sequences were enriched not only in exons but also in introns (Figure 3A). Results of NAT10 RIP-seq in another study (Zhang et al., 2021) also showed a peak within the intronic sequence of COL5A1 mRNA, and ac4C promoted the stability but not translation efficiency of COL5A1. Based on these results, it is established that NAT10 binds to intronic sequences, but it is not known whether NAT10 binding results in ac4C production in intronic regions of pre-mRNAs. It is possible that ac4C modification exists within pre-mRNAs and regulates gene expression by influencing stability rather than translation efficiency. Besides, non-coding RNAs like long non-coding RNA (lncRNA) can be produced by intronic and intergenic sequences. It is reported that the m6A writer METTL14 methylated and down-regulated lncRNA XIST in cancer cells (Wang et al., 2021). Similarly, ac4C may also modify and regulate non-coding RNAs, although its existence within non-coding RNAs have not been reported. At present, our knowledge of ac4C existence and functions is still limited. Further research and more data will help us get a clearer picture.

ac4C was first identified as an mRNA modification by Arango et al. (2018). As a recently identified mRNA modification, the regulation of ac4C remain largely unknown. ac4C production within mRNA, tRNA and 18s rRNA is catalyzed by NAT10 or its homologs across different species (Chimnaronk et al., 2009; Ito et al., 2014; Sharma et al., 2015; Arango et al., 2018). Although it is established that ac4C promotes mRNA stabilization and translation, the precise mechanisms by which ac4C modulates downstream cellular activities remain to be elucidated. Proteins specifically binding to ac4C modification have not been identified yet.

In the present study, we identified a series of proteins specifically binding to acetylated RNA probes by mass spectrometry and predicted a list of RBPs potentially binding to ac4C motifs by an online algorithm. Combined analysis demonstrated that TBL3 is a potential ac4C-binding protein. TBL3 is an 89-kDa protein, mainly located in the nucleus and known as an RNA-binding protein, which is involved in endonucleolytic cleavage in 5′-end of rRNA and rRNA processing (Gaudet et al., 2011; Castello et al., 2012). It is one of the core components in human small subunit processome, a 2.2MDa ribonucleoprotein complex, where rRNA precursors are processed and ribosomes are assembled and maturated (Wada et al., 2014). TBL3 contains WD40-repeats, which are conserved domains acting as structural platforms for macromolecular interactions (Wada et al., 2014). It is also reported that TBL3 affects the stem cell-associated functions, including tissue homeostasis, regeneration, and stem cell maintenance in both invertebrates and vertebrates (Labbe et al., 2012). Our results suggest that TBL3 preferentially binds to ac4C RNA. Although we confirmed their preferential binding by RNA pulldown using RNA probes of certain sequences, we cannot apply our results to other sequences. We also demonstrated TBL3 expression in oocytes by immunoblot. However, it remains to be determined whether TBL3 directly binds to ac4C sites or whether TBL3 mediates ac4C-exerted functions in oocytes. Therefore, further research is needed to clarify whether TBL3 interacts with the modification directly regardless of sequences, to determine the critical domain for binding, to explain the biological significance of TBL3 binding to ac4C RNA, and to investigate the role of TBL3 in oocytes.

As has been stated, our results suggest that NAT10-mediated ac4C modification is an important regulatory factor during oocyte maturation *in vitro*, by regulating genes associated with nucleosome assembly, chromatin silencing, chromatin modification and cytoskeletal anchoring. Furthermore, TBL3 is a potential ac4C-binding protein and may mediate downstream cellular activities. Still, we acknowledge some limitations to our study. First, due to technical limitations, we were unable to perform NAT10-RIP or acetylated RNA immunoprecipitation (acRIP) in oocytes. Thus, the dysregulated genes were identified in HEK293T cells, which may not reflect the conditions in oocytes. Second, since ac4C modification regulates gene expression by influencing mRNA degradation or translation, the transcript level of a certain regulated gene may remain constant. Transcriptome alone is not sufficient to illustrate the influence exerted by ac4C. We are searching for techniques revealing both transcriptome and ribosome-profiling of a single oocyte, which will enable us to illustrate the exact impact that ac4C exerts on a certain gene. Last, our data suggest that TBL3 preferentially binds to ac4C RNA probes, but cannot determine whether TBL3 binds to ac4C modification directly. More studies are required until it can be confirmed as an ac4C reader.

## Conclusion

In this study, we have documented the down-regulated expression of ac4C and NAT10 during meiotic maturation of mouse oocytes. NAT10 knockdown resulted in ac4C reduction and impaired mouse oocyte maturation *in vitro*. These results indicated that NAT10-mediated ac4C modification plays a critical regulatory role in oocyte meiotic maturation. In addition, we identified TBL3 as a potential ac4C-binding protein, through which ac4C may exert its function in downstream biological activities. Still, further research is required for confirmation.

## Data Availability Statement

The data presented in the study are deposited in the GEO database (https://www.ncbi.nlm.nih.gov/geo/, accession number: GSE179429) and the PRIDE database (http://proteomecentral.proteomexchange.org/cgi/GetDataset, accession number: PXD027014).

## Ethics Statement

Animal experiments were approved by the Committee on the Ethics of Animal Experiments of The Sixth Affiliated Hospital, Sun Yat-sen University (ethical approval number: IACUC-2020120401).

## Author Contributions

XL funded this study. CZ, HZ, and XL conceived this study. YX, QG, YZ, JH, TW, JL, and QL performed the experiments. YX and CZ analyzed the data. YX wrote the manuscript. CZ and XL revised the manuscript. All authors contributed to the article, reviewed the manuscript, and approved the submitted version.

## Conflict of Interest

The authors declare that the research was conducted in the absence of any commercial or financial relationships that could be construed as a potential conflict of interest.

## Publisher’s Note

All claims expressed in this article are solely those of the authors and do not necessarily represent those of their affiliated organizations, or those of the publisher, the editors and the reviewers. Any product that may be evaluated in this article, or claim that may be made by its manufacturer, is not guaranteed or endorsed by the publisher.

## Footnotes

1
https://david.ncifcrf.gov

2
http://service.tartaglialab.com/page/catrapid_group

3
https://www.ncbi.nlm.nih.gov/geo/

4
https://www.ncbi.nlm.nih.gov/pubmed

## Supplementary Material

Supplementary Figure 1 Representative images of negative control staining for immunohistochemistry (without the primary antibody). **(A)** Negative controls of untreated mouse oocytes. Fluorescence exposure time: GV, 300 ms; MII, 1.9 s. **(B)** Negative controls of transfected mouse oocytes. Fluorescence exposure time: 300 ms. Scale bar = 50 μm. GV, germinal vesicle. MII, metaphase II.
