# Supplementary figures and images for: NAT10-Mediated N4-Acetylcytidine of RNA Contributes to Post-transcriptional Regulation of Mouse Oocyte Maturation in vitro
Source: Front Cell Dev Biol. 2021 Jul 30;9:704341. doi: 10.3389/fcell.2021.704341 (PMC8363255; doi:10.3389/fcell.2021.704341)

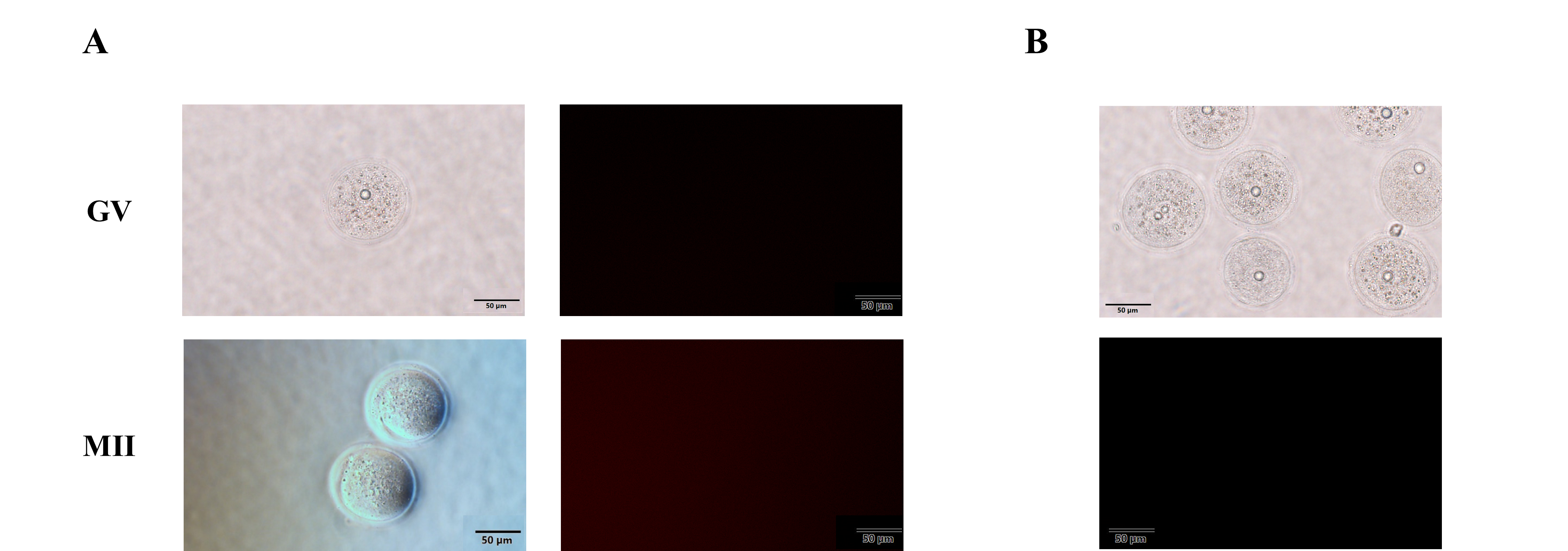

Supplement: Supplementary Figure 1 — Representative images of negative control staining for immunohistochemistry (without the primary antibody). (A) Negative controls of untreated mouse oocytes. Fluorescence exposure time: GV, 300 ms; MII, 1.9 s. (B) Negative controls of transfected mouse oocytes. Fluorescence exposure time: 300 ms. Scale bar = 50 μm. GV, germinal vesicle. MII, metaphase II. [file Image_1.TIF]
